# Online assessment standard setting for multiple choice questions

**DOI:** 10.5116/ijme.5715.3481

**Published:** 2016-05-05

**Authors:** Sami Shaban, Margaret Elzubeir, Mohammed Al Houqani

**Affiliations:** 1 Medical Education Department, College of Medicine and Health Sciences, United Arab Emirates University, Alain, UAE; 2 Internal Medicine Department, College of Medicine and Health Sciences, United Arab Emirates University, Alain, UAE

**Keywords:** Online assessment, standard setting, multiple choice questions, United Arab Emirates

## Introduction

Standard setting of assessment questions has matured as a psychometric practice since the 1980s^1^ and is now a common practice in Medical Education.^2^ This is done to ensure that a fair, defensible cutoff score or pass mark is reached for each assessment paper.^3^ Standard setting is thus a policy decision as standards are not a natural phenomenon waiting to be discovered,^4^  neither does the process signify scientific precision. Nevertheless, in order to be defensible standard-setting must be procedurally credible.

At the College of Medicine and Health Sciences (CMHS), United Arab Emirates (UAE) University, the Modified Angoff^5^ procedure of standard setting has been adopted as applicable to all high stakes and end course MCQ examinations since 2014. This test-centered, criterion referenced method of standard setting is one of the most studied and widely used procedures for high stakes examinations.^4^

At the CMHS, following a period of question vetting, the procedure was initially conducted in face-to-face meetings of appropriate faculty members with subject expertise. In this exercise judges make independent estimates of the proportion of minimally competent candidates that would be expected to answer each item correctly. Where there is a large variation in estimates, judges then discussed discrepancies and revised estimates. Depending on length of discussion and necessity to refine previously vetted questions, the procedure often took an average of three to six hours to standard set an examination of 100 MCQs. In the case of high stakes examinations, the procedure could take several times that amount of time.

Although this process had its benefits in further vetting of questions, useful discussion and feedback between faculty members about their understanding of the task, issues surrounding the minimally competent candidate, etc., it was also critiqued by faculty as arduous and time consuming for busy medical subject matter experts. Availability of new technologies have however facilitated evolution of methods and processes of test administration and setting cut scores to meet challenges posed by real and perceived inadequacies of existing processes.^6^^,^^7^

The purpose of this paper is to describe how we implemented an online standard setting procedure which significantly reduces the amount of time needed to standard set examinations.

### Implementation

We set out to automate the process of standard setting at CMHS. Relying on an online, secure Assessment Management System (AMS)^8^ where the questions are stored safely by the Medical Education Department, vetted and standard set by faculty, and delivered securely to students. The system is developed in-house using a secure internal website running ASP and MS SQL Server. The system involves two types of users, judges who are faculty members able to make a judgment of the appropriate cutoff mark for each question, and coordinators who are in charge of courses and associated examinations.

The system was used at CMHS as a pilot for several course final examinations and is now used for all less high-stakes examinations with judges making judgments using their office computers while entering comments on questions that warrant discussion. It is also used for high-stakes examinations in face-to-face meetings with judges using handheld devices to access the questions, have any needed discussion, and then enter their judgments in their handheld devices. The resulting cutoff average, standard deviation, and histogram are displayed on the handheld device and an overhead projector and modification of cutoffs can be performed if necessary.

The judges can read the question clearly, make a judgment on the percent of minimally competent students who should answer the question correctly, enter that percent easily, and add a comment about the question if they wish. Judges can be assigned, removed or emailed by the coordinator. Completion percent and average cutoff for each judge are clearly shown and comments can be displayed.  Questions that have a cutoff standard deviation among judges of more than 20% are marked so that they may be reviewed in a short meeting along with the questions and comments from judges. Each question that has been standard set display the average cutoff, standard deviation and number of judges along with the histogram of cutoffs and all judges’ comments on that question.  The assessment analysis shows, question by question, percentage of options chosen, point biserials for correct options, and the difference between students' correct option percentage and judges' cutoffs. This provides an indication regarding the accuracy of judges' cutoff estimations.

We feel that we have uncovered some novel ideas in Medical Education standard setting which warrant further research. For example, what is the appropriate cutoff standard deviation to identify questions that need review (we use 20% as rule of thumb)? And, what classifies a reasonable judgment in comparison to students’ correct response (we use judges' average within ±20 of students' correct response as another rule of thumb)?

Finally, similar to other researchers, we are confident that in time online standard setting methods will proliferate, but believe this will only happen when these new ideas are addressed effectively. Furthermore, as in the case of most standard setting research, we cannot state with confidence that the procedure described here is widely generalizable. We can nevertheless, attest to the overwhelming positive feedback received from standard setters in our institution and recommend that other institutions faced with similar constraints necessitating consideration of an alternative approach, at least pilot online standard setting.

## Conclusions

There are important advantages and challenges to conducting standard setting in a virtual environment. Using an online, secure system for standard setting of assessments can be an efficient way to collect judgments of experts regarding the appropriate pass mark of questions. User feedback has been overwhelmingly positive as the system is secure, user-friendly, and saves a great deal of meeting time since meetings are limited to reviewing only questions with comments or questions with cutoff standard deviations of more than 20%. The challenges include the fact that traditional meeting logistics (meeting room, catering, etc) are replaced by technological logistics. Because participants have to access test content when making judgments, security concerns may be heightened and administrators have to place considerable trust in participants. Finally, the orientation and training process cannot be bypassed and must be conducted in the traditional face-to-face manner to ensure participants can effectively engage in the on-line process.

### Conflicts of Interest

The authors declare that they have no conflict of interest.
